# Quantified Soccer Using Positional Data: A Case Study

**DOI:** 10.3389/fphys.2018.00866

**Published:** 2018-07-06

**Authors:** Svein A. Pettersen, Håvard D. Johansen, Ivan A. M. Baptista, Pål Halvorsen, Dag Johansen

**Affiliations:** ^1^School of Sport Sciences, UIT The Arctic University of Norway, Tromsø, Norway; ^2^Department of Computer Science, UIT The Arctic University of Norway, Tromsø, Norway; ^3^ForzaSys AS, Oslo, Norway; ^4^Simula Research Laboratory, Oslo, Norway

**Keywords:** player load, athlete quantification, GPS tracking, LPM tracking, wearables, player monitoring

## Abstract

Performance development in international soccer is undergoing a silent revolution fueled by the rapidly increasing availability of athlete quantification data and advanced analytics. Objective performance data from teams and individual players are increasingly being collected automatically during practices and more recently also in matches after FIFA's 2015 approval of wearables in electronic performance and tracking systems. Some clubs have even started collecting data from players outside of the sport arenas. Further algorithmic analysis of these data might provide vital insights for individual training personalization and injury prevention, and also provide a foundation for evidence-based decisions for team performance improvements. This paper presents our experiences from using a detailed radio-based wearable positioning data system in an elite soccer club. We demonstrate how such a system can detect and find anomalies, trends, and insights vital for individual athletic and soccer team performance development. As an example, during a normal microcycle (6 days) full backs only covered 26% of the sprint distance they covered in the next match. This indicates that practitioners must carefully consider to proximity size and physical work pattern in microcycles to better resemble match performance. We also compare and discuss the accuracy between radio waves and GPS in sampling tracking data. Finally, we present how we are extending the radio-based positional system with a novel soccer analytics annotation system, and a real-time video processing system using a video camera array. This provides a novel toolkit for modern forward-looking soccer coaches that we hope to integrate in future studies.

## 1. Introduction

Over the last decade, we have witnessed the emergence of a myriad of wearable devices and sensors for quantification of sport and physical activity. These are frequently touted as a game changer and a key for future development of many sports. Key sport governance organizations like Fédération Internationale de Football Association (FIFA), with its 265 million members in various local clubs world-wide (Kunz, 2007), have already approved use of wearables and Electronic Performance and Tracking Systems (EPTSs) in official matches. This has undoubtedly accelerated research and development of athlete quantification technology. Training and matches are already being impacted. For instance, it is believed that the German national soccer team used wearable technology to profile the players, and with these statistics, coach Joachim Low made the crucial substitute of Mario Götze who scored the winning goal in the world cup final in Brazil 2014.

Although such success stories certainly do exist, the general usefulness of athlete quantification technologies has several shortcomings. The aim of this paper is to highlight some of the challenges we encountered when using positional data as part of research and team development, and to suggest other promising data sources. Our main observation is that athlete quantification systems are often inhibited by questionable validity of acquired data. We argue that by combining data from multiple systems, some of the shortcomings of existing positional tracking systems can be detected and perhaps avoided. All data in this report was collected from autumn 2011 until spring 2017. All participants have given their written informed consent, and the project has been given institutional approval.

## 2. Tracking using LPM (radio signals) and GPS in a professional football club

Football is an open-loop sport, and it is important to emphasize the need for more research to develop our understanding of valid indications of physical match performance and competitive success (Carling, 2013). Toward that end, the athlete quantification technologies deployed in our research facilities at Alfheim Stadium is already generating important insight. At Alfheim Stadium, there has been a substantial development and use of various tracking technology, including multiple camera semi-automatic systems, Local Position Measurement (LPM) systems, and GPS systems, each capable of quickly recording and storing data about team players. We have to a large extend moved away from GPS based technology, which has traditionally been the preferred choice by clubs to quantify training load of team-sports athletes, both during training and matches (Aughey, 2010).

An alternative to GPS based systems, are those based on LPM radio signals. Unlike GPS systems, where devices are passive receivers of signals from overhead satellites, LPM systems work by having the wearable emit signals to local receivers, which do the actual triangulation. Our experience is that LPM systems have better accuracy than GPS-based systems. In our case, we have several years of experience with positional tracking using the stationary LPM system: ZXY Sport Tracking System by ChyronHego (Trondheim, Norway). This system is based on using the 5.0 GHz Industrial, Scientific, and Medical (ISM) radio band for communication and signal transmissions. With ZXY, each player wears a belt with a transponder placed at his lumbar (Pettersen et al., 2014), and there are six stationary sensors placed at the stadium perimeter. The stationary sensors compute the position data for each belt by advanced vector based processing of the received radio signals. The processing system in each stationary sensor enables direct projection of the player's positions on the field without having to exchange data with other sensors. Multiple receivers are still required to cover the entire field and to avoid occlusions. The default resolution is fixed to 20 Hz for each belt. Data is stored in the system's internal database and can be exported as comma separated values files.

To quantify the accuracy difference of GPS technology compared to LPM systems, we performed two studies, as will be described next.

### 2.1. Study 1 and study 2: GPS vs. LPM-tracking

In Study 1 (2011), we instrumented 6 high-level female players (weight 59.6 ± 6.8 kg, height 171.5 ± 4.2 cm) with both GPS and LPM tags and instructed them to perform the Copenhagen Soccer Test for Women (CSTw). Each player ran the CSTw course 18 times, simulating a match and accumulating a distance of 10,331 m (Bendiksen et al., 2013). Each player wore two GPS tags from the GPSport SPI-ProX1 5.0 Hz system in a vest on their upper body, and two ZXY tags placed in a small belt near the lumbar spine. Having multiple tags enables us to measure both the inter and the intra reliability of the systems.

The average distance covered was measured by SPI-ProX1 (12 tags on 6 players) to 11,668 ± 1,072 m with a CV value of 6%, while ZXY (14 tags on 7 players) measured the distance to 10,204 ± 103 m with a CV value of 1%. For High Intensity Runs (HIRs) (>16.0 km h^−1^), the values were 612 ± 433 m with a CV value of 37.4% and 1238 ± 38 m with a CV value of 3.1%, respectively.

In the intra reliability test, the measured discrepancy between the two tags placed on the same player ranged between 800 and 2,071 m using SPI-ProX1 and 25–290 m using ZXY. Our observation that the SPI-ProX1 system seems to measure higher values for total distance covered is further supported by an experiment where 19 players of two junior elite teams were equipped with both ZXY and SPI-ProX1. The average distance covered was measured by SPI-ProX1 to 10,805 ± 847 m, while ZXY measured the distance to 9,891 ± 974 m (Johansen et al., 2013).

**Figure 1:**
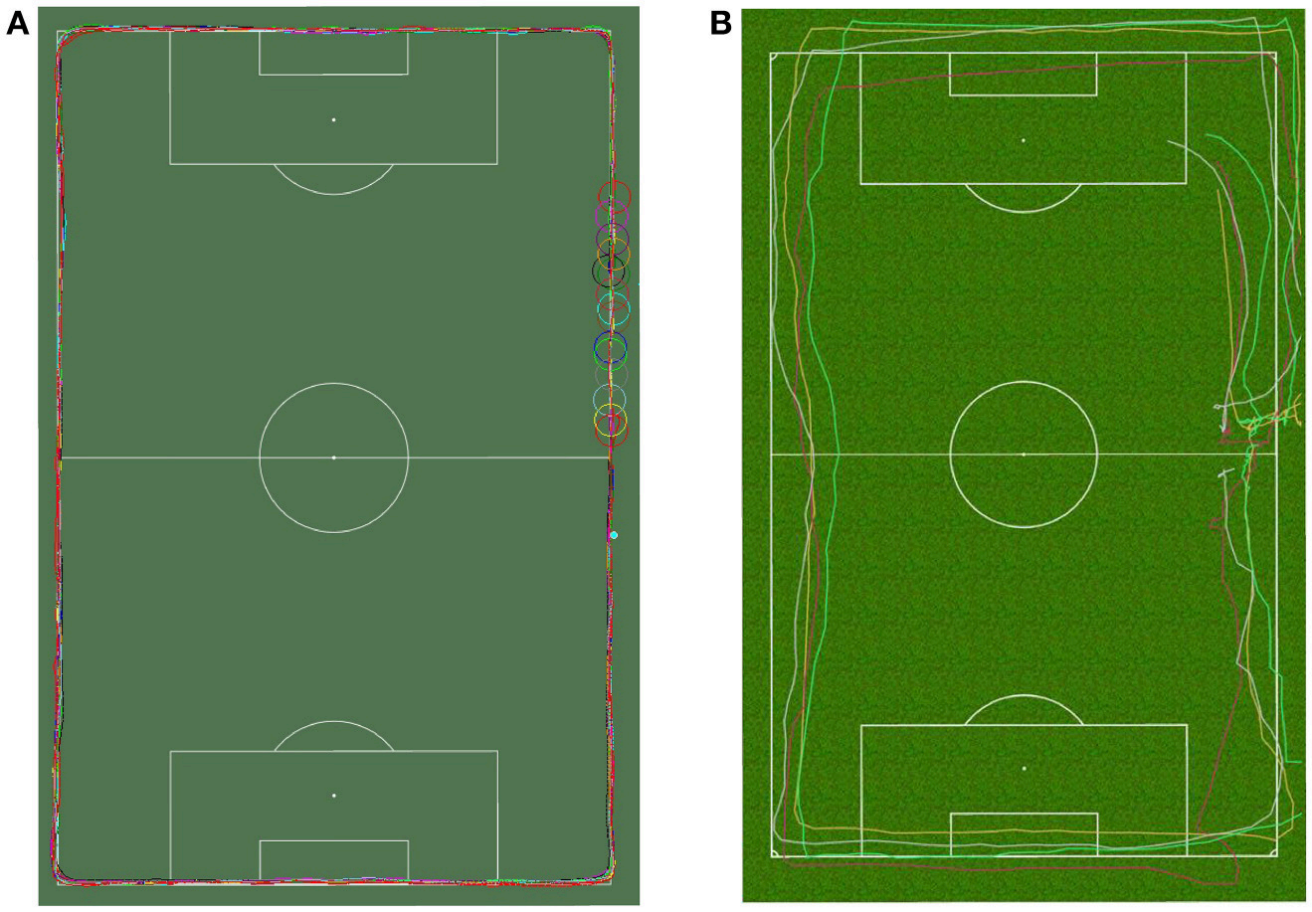
Comparison of tracking technologies in Study 2 for 12 players running at the side and endlines of the pitch at Alfheim Stadium. **(A)** LPM tracking tracking results (ChyronHego ZXY, 12 players shown. **(B)** GPS tracking results (Polar Team Pro, 5 of 12 players shown. The figure shows movement after the experiment cutoff.

In Study 2 (2016), 12 male youth elite players (weight 64.2 ± 8.2 kg, height 176.0 ± 6.7 cm) were instructed to jog clockwise around the pitch at Alfheim Stadium, following the side and end-lines of the pitch. All players wore both the Polar Team Pro 10 GHz GPS system (Kempele, Finland) and the ZXY system. The GPS tags were connected to the anterior part of the chest by a elastic chest strap. Figure 1B shows the recorded positional information for both Polar and ZXY. (The Polar system could not plot more than five players per figure.) As can be seen in the figures, players were not capable of performing 90° turns in the corners, which is to be expected. The GPS tracks in Figure 1B can clearly bee seen to deviate significantly from the actual trajectory of the players, while the tracks shown in Figure 1A much more closely follow the lines. A similar effect was also observed by Buchheit et al. (2014).

Next, seven of the twelve players were selected to complete a training session. With statistical significance levels obtained by Paired *T-*test, sprint performance (>25.2 km h^−1^) was measured lower by ZXY 55.3 ± 7.3 m compared to Polar Team Pro 70.0 ± 12.9 m (*P* > 0.05). HIR and number of accelerations (≥2 m s^−2^) showed an inverse tendency with higher values 222.8 ± 77.8 m and 100.9 ± 19.9 counts vs. 164.4 ± 54.9 m and 81.0 ± 15.9 counts (ns). All tracking generated raw data was loaded into Microsoft Excel, where statistical procedures were executed.

It could be speculated that the GPS signal reception at Alfheim Stadium is poor. However, the stadium does not have an overhanging roof, nor are there any nearby high buildings that obscures the sky. A few 9 m high stands are located 9.3 m behind the sidelines, but we do not suspect these to interfere with the GPS signal. Measurement accuracy may still be reduced by atmospheric conditions such as clouds and fog. A more plausible explanation is perhaps the stadium's arctic location at 69.65° north. The inclination of GPS satellite orbits is approximately 55° (north or south), so that no satellites have been directly overhead during our tracking sessions (Langley, 1999). High error rates have, however, been reported elsewhere for inter-unit reliability across different GPS models (Jennings et al., 2010; Castellano et al., 2011). A stationary reference GPS receiver can improve accuracy by averaging its position over time. As long as such a reference receiver detects the same satellite signals as the wearable GPS receiver, it can send correction data. In the northern areas, GPS based solutions that also communicate with the Russian Global Navigation Satellite System (GLONASS) system should also be considered as these generally provide better precision here. Still, ours and Stevens et al. (2014) findings indicate superior accuracy in Local Position Systems (LPS) compared to GPS. It remains unclear to what extent the inherent accuracy limitation in the GPS system limits its usefulness for athlete quantification.

Although the CSTw has a 10,331 m preset course that the players should follow, some discrepancies in the measured distance are to be expected. Even small deviation of the sensor device from the set trajectories of the test, like the player leaning in the turns of the course, will impact the measurements and adds up throughout the test. However, the high meter values in relation to the course length and in addition the large CV between units of the SPI-ProX1 system suggest that the results should be interpreted with caution.

Using an absolute sprinting or high-velocity threshold for all athletes in a team does not account for individual genetic or physiological differences. The same external load calculated by an acceleration, HIR, or sprinting threshold for two athletes could represent a different internal load based on individual characteristics (Impellizzeri et al., 2004). Positive and negative accelerations are metabolically demanding and often do not elicit velocities defined as HIR or sprint (Osgnach et al., 2010). The starting velocity is critical when measuring accelerations or decelerations, the metabolic cost of changing speed more than 2.0 m s^−2^ is much larger at a starting speed of 5.0 m s^−1^ compared to 1.0 m s^−1^. In addition, quantification of these variables is dependent upon the validity and reliability of athlete tracking systems.

An alternative may be individual thresholds for external load expressed relative to maximum speed attained during sprint testing. An individualized approach of arbitrarily derived velocity thresholds may benefit the training prescription for players, but will limit comparisons with other teams and leagues. Limited research exists on how to individualize accelerations, which are energy demanding, and therefore, we will have limited information on total external load even with individualized speed zone limits (Sweeting et al., 2017).

### 2.2. Study 3: high intensity activity in training vs. match

In Study 3 (2017), 5 players (age 25.2 ± 4.0, height 178.4 ± 5.0 cm, weight 75.2 ± 6.6 kg) were randomly selected from 5 different playing positions: central back, full back, central midfielder, wide midfielder, and central forward. The players were tracked in 5 consecutive in-season training sessions (microcycle) and in one official home match. Distances and number of HIR and sprints were compared (Table 1). We observed large discrepancies in high-intensity activities between trainings in the microcycle and match. As shown in Table 1, we have recorded substantial underload in HIR and sprint for most players during the training week compared to macth. Following the *principle of overload*, this indicates that the format of the small side games does not elicit the sufficient amount of HIR and sprint, with exception of the central forward position in the team's style of play. Practitioners should be aware of and take into consideration how different pitch size and number of players dictate the external and internal training load.

**Table 1:** High-intensity actions (HIRs and Sprints) and number of appearances (counts) and/or meters for five training sessions, compared to an official match in five players in different positions.

	**High Intensity Runs (HIRs)**						**Sprints**					
	**Count**		**%**	**Dist (m)**		**%**	**Count**		**%**	**Dist (m)**		**%**
	**Match**	**Train**.	**Match**	**Match**	**Train**.	**Match**	**Match**	**Train**.	**Match**	**Match**	**Train**.	**Match**
CB	35	38	109	560	327	59	8	7	88	112	58	52
FB	44	54	123	835	694	83	13	11	85	183	104	57
CM	60	56	93	1305	698	53	16	4	25	259	67	26
WM	49	60	122	1032	559	54	18	10	56	228	84	37
CF	49	54	110	851	705	83	10	15	150	103	153	149

*CB, Center back; FB, Full back; CM, Center midfield; WM, Wide midfield; CF, Center forward*.

*The difference (% match) correspond to the total value of the training week compared to the match. The value of the match is considered as 100%. Example from a normal microcycle (5 training sessions between two official matches)*.

From a training load perspective, the large intra/inter unit differences in tracked distance described in section 2 can also have significant practical implications for an athlete across a longitudinal period, which questions meaningful interpretation of the data. For within-athlete longitudinal monitoring, we therefore recommended that practitioners assign a specific device to each athlete. To appropriately detect changes in physical performance, researchers must also account for match-to-match variation and device reliability. Any possible interference between co-located devices has to our knowledge not yet been fully explored. Nevertheless, developing a device including algorithms describing position-specific match demands might be useful to control training load in relation to match demands. By integrating information about training content, load periodization, and fatigue status we can provide real-world insight into optimal approaches for player preparation.

## 3. Perspective

The studies described above indicate that existing positional technologies do not guarantee an accurate measurement of player locomotor activities. We are therefore experimenting with two specific supplemental data sources that we plan to integrate in future studies: one based on video and one based on self-reporting.

### 3.1. Full-stadium video coverage

Video of player actions are generally considered a useful tool for soccer analytics. Videos have traditionally been obtained from the following three sources: professional TV broadcasts, hand-held cameras, or fixed arena cameras. Unfortunately, these sources are either not available for practices, too personnel demanding, or too costly. More importantly, none of these solutions provide a sufficient high-resolution coverage of all players throughout a session. Our solution was to develop the Bagadus (Stensland et al., 2014) video system.

Bagadus consists of multiple small shutter and exposure synchronized cameras that record a high-resolution video of the soccer field. The cameras are set in a circular pattern; pitched, yawed, and rolled to look directly through a point five cm in front of the lenses, minimizing the parallax effect. Combined, the cameras cover the full pitch with sufficient overlap to identify common features necessary for camera calibration and image stitching to generate a panorama video.

Bagadus video playback can switch between streams delivered from the different cameras, either manually by selecting a camera, or automatically following players based on sensor information. It can also play back a panorama video stitched from the different camera feeds. Using the panorama video, a virtual view can also be extracted (Gaddam et al., 2015), for instance to automatically follow one particular player (Gaddam et al., 2014).

### 3.2. Video indexing with rich metadata

Many elite soccer clubs spend much time on manual labor-intensive post-game analysis by carefully watching full-length recordings of the game. By enriching video archives with time-synchronized metadata from external sensors, Bagadus enables a much more efficient video retrieval and summarizing experience, reducing the time needed for coaches to locate relevant video segments. At Alfheim Stadium we found positional data from ZXY particularly useful as it enables Bagadus to track individual players and generate on-the-fly video summaries based on player or group formation and trajectories. For instance, a video summary of all situations where a particular player sprints toward his own goal, or all situations where the midfielder is in the mid-circle (Mortensen et al., 2014).

In addition to positional data, we have developed an annotation system (Johansen et al., 2012; Stensland et al., 2014) for use *during* matches to tag important events with metadata as they occur. A key design principle for this system was minimizing deployment effort and hardware investments. Mobile devices like smartphones and tablets are as such ideal platforms as they are highly available, mostly Internet connected, and provide sufficient computational resources. In combination with an tile-based interface optimized for fast input, the average annotation time was cut down to less than 3 seconds (Johansen et al., 2012) while operated on the field. The registered events are time-aligned with the video and stored in an analytic database, immediately available for use by the video retrieval system. This enable video-based team or individual feedback in the locker room during half time, or after practice.

### 3.3. Individual subjective reports

We have also implemented a player monitoring system PMSys: a self-reporting system^1^ for mobile devices, which enables monitoring of individual phenotypic parameters through repeated questionnaires that the players answer on their own mobile phones.

Having regular reports from all team members is a key goal for PMSys. As such, a key design requirement was support on all smart-phone platforms (e.g., iOS and Andoid) in use by team members. To reduce the costs of multi-platform support, we opted to develop PMSys as a hybrid-mobile application based on the Ionic 2+ Framework^2^. Recent versions of the framework generate applications that look and feel similar to native ones, and earlier performance and appearance disadvantages are mostly mitigated. PMSys is currently deployed in Google Play for Android devices, and in Apple's iTunes store for iOS devices. The mobile application provides graphical visualization feedback, which gives the player a timeline overview.

In addition to the smart-phone app, we also constructed a web-portal that team coaches can use to analyze and present data. The portal is constructed with the coaches in mind, providing several tools and plots for teams and individual players. In combination with the web portal and mobile application, we have implemented our own communication service between the mobile phone and the web portal, allowing a coach to send push-messages directly to a player's mobile phone. A key feature of PMSys is the ability for coaches to schedule future and repeated push-messages.

Our experience with PMSys Athlete Self-Report Measures (ASRM) at Alfheim, is that education and feedback is of utmost importance to maintain daily usage. The scope of education should include why an ASRM should be used, the purpose of the questions asked, and who is analysing the data. Education should emphasize that results are to be used for the player benefit, and not to their detriment. Feedback should consist of daily interactions and reminders pushed directly to the users device, showing what action is taken in response to reported data. During the season, the generated daily wellness reports may form the basis of the regular conversations between coaching staff and players. Engagement of staff, especially in the implementation process, is essential (Saw et al., 2015), with particular emphasis on the need for a key-staff member to oversee the day-to-day responses and be able to analyze and interpret the ASRM.

By complimenting GPS and LPM positional data, like the ones we have used in our previous studies, with data from video and self-reporting tools, we hope to better predict injury or reduced performance for a player. The extended data sources are in particular interesting when considered as additional input to modern machine learning algorithms.

## Ethics statement

The study is approved by the Norwegian Centre for Research Data and the players have given their written informed consent to participate.

## Author contributions

SP: data collection, in charge of the writing process; HJ, IB, PH, and DJ: data collection, manuscript writing.

### Conflict of interest statement

We hereby declare that PH is employed in a part-time position at ForzaSys AS. The remaining authors declare that the research was conducted in the absence of any commercial or financial relationships that could be construed as a potential conflict of interest.

## Footnotes

^1^PMSys, http://forzasys.com/pmsys.html.

^2^https://ionicframework.com/
